# Medication Timing Errors for Parkinson's Disease: Perspectives Held by Caregivers and People with Parkinson's in New Zealand

**DOI:** 10.4061/2010/432983

**Published:** 2009-11-05

**Authors:** Stephen Buetow, Jenny Henshaw, Linda Bryant, Deirdre O'Sullivan

**Affiliations:** ^1^Department of General Practice and Primary Health Care, University of Auckland, Auckland 1142, New Zealand; ^2^Parkinson's New Zealand, Wellington 6142, New Zealand

## Abstract

*Background*. Common but seldom published are Parkinson's disease (PD) medication errors involving late, extra, or missed doses. These errors can reduce medication effectiveness and the quality of life of people with PD and their caregivers. *Objective*. To explore lay perspectives of factors contributing to medication timing errors for PD in hospital and community settings. *Design and Methods*. This qualitative research purposively sampled individuals with PD, or a proxy of their choice, throughout New Zealand during 2008-2009. Data collection involved 20 semistructured, personal interviews by telephone. A general inductive analysis of the data identified core insights consistent with the study objective. *Results*. Five themes help to account for possible timing adherence errors by people with PD, their caregivers or professionals. The themes are the abrupt withdrawal of PD medication; wrong, vague or misread instructions; devaluation of the lay role in managing PD medications; deficits in professional knowledge and in caring behavior around PD in formal health care settings; and lay forgetfulness. *Conclusions*. The results add to the limited published research on medication errors in PD and help to confirm anecdotal experience internationally. They indicate opportunities for professionals and lay people to work together to reduce errors in the timing of medication for PD in hospital and community settings.

## 1. Introduction

Medication-related errors are common [1], but not often reported, in the treatment of Parkinson's disease (PD), a chronic and disabling neurodegenerative disorder whose prevalence increases with age [2] and is likely to rise with population aging. These errors can include the timing of medication ingestion for PD [3]. Irregular timing, especially of L-Dopa and in later stages of the disease [4], can adversely affect those who have PD, as well as their informal caregivers [5]. Late, extra or missed doses can reduce medication efficacy—losing health gain, contributing to motor and non-motor fluctuations [6] and impairing function and quality of life.

Timing errors for PD can take place in diverse health settings. The need for people with PD to receive the right medication at the right time in hospitals and care homes underpins the ‘‘Get it on time” campaign of Parkinson's Societies internationally [7, 8]. Also common in community settings are the missed and mistimed doses attributable in whole or part to the (in) actions of people with PD and their caregivers [9, 10]. This nonadherence has been reported to result mainly from being ‘‘too busy/forget” or having ‘‘left home without (the) drug” [9]. 

Approaches that health professionals use to identify and measure their timing errors can include error reports, record review, clinical surveillance, and observations of care [11]. However, people with PD, and their caregivers, can also observe formal and informal care [12]; disclose their own timing mistakes [13]; and share their error-related concerns about professionals' timing of medication for PD. Formal safety assessments have tended to ignore lay errors and lay perspectives [13]. Yet these perspectives link strongly to lay satisfaction with the health care experience [14]. Also, they “often reveal how well a hospital system is operating and can stimulate important insights into the kinds of changes that are needed to close the chasm between the care provided and the care that should be provided” [15, page 33]. Health professionals need to be aware of, and responsive to, these perspectives if those who are most directly affected by PD are to trust them and engage in programs to identify, understand and manage timing errors [13, 14]. 

The muted voices of patients and caregivers have contributed to a paucity of research evidence on the nature and significance of the factors that can contribute in hospital and community settings to actual or perceived errors around medication timing for PD. Therefore, this paper seeks to explore lay experience, understanding and perspectives of these factors. It addresses this aim as part of our larger study of medication-related errors in PD in New Zealand, whose health system is funded from general taxation and includes free or subsidised health care for those suffering from chronic medical conditions. Ethics approval was obtained from New Zealand's multi-region health and disability ethics committee.

## 2. Materials and Methods

### 2.1. Sampling

A sample was purposively selected of individuals with PD, who were sometimes represented by a proxy of their choice, throughout New Zealand during 2008–2009. These volunteers had read an article published by the researchers in the June 2008 issue of Parkinson's New Zealand's quarterly magazine (The Parkinsonian), or had been invited by one of its field officers to consider participating in the study. The volunteers had consented in writing to give an interview and had reported, on a screening questionnaire, that they, or the person on whose behalf they were speaking, (a) had PD and (b) had “one or more stories to tell about my experience of a possible mistake relating to my medicine for Parkinson's.”

### 2.2. Data Collection

Twenty semistructured, personal interviews were conducted by telephone. All the interviews were conducted in English, mostly by JH but also SB. Participants were asked to tell the interviewer about occasions when they perceived having experienced possible mistakes with with the medication prescribed for PD. The interviewer explored, among other things, what the participants perceived to have caused these mistakes. Each interview was audio-recorded, with participants' consent.

### 2.3. Data Analysis

Transcripts of the interviews were imported into QSR NVivo, a software programme for managing and supporting the analysis of qualitative data. A general inductive analysis of the data [16] was then used to identify core meanings consistent with the study aim. This approach involved closely reading and coding all the transcripts to generate and refine emergent themes. The other authors took the role of sceptical peer reviewers before the participants were sent the analysis to check.

## 3. Results

Table 1 summarises the sample of 20 participants. Thirteen had PD. Seven were proxies who spoke on behalf of a person with PD. The interviews, therefore, referred to 20 people with PD, of whom 14 were male and 14 were aged at least 65 years. Sixteen were reported to have PD of at least moderate severity, and to have had a diagnosis of PD for 11 years on average (standard deviation (*s*) = 5.6). For 13 people with PD, the hospital was where a perceived error with PD medication took place. The duration of the interviews averaged 25.9 minutes (*s* = 10.9). Two potential participants were excluded because they reported having atypical Parkinson's: Lewy Body Disease and Multiple System Atrophy, respectively. Five salient themes were identified to help explain possible timing adherence errors by people with PD, their caregivers or professionals. Each theme is described in turn.

### 3.1. Abrupt Withdrawal of PD Medication

Medication for PD is not given on time when it is stopped deliberately and abruptly. P12 described how the benign hallucinations of her husband with PD worsened in hospital. She attributed this sudden change to the morphine administered for his two broken hips. However, the hospital was said to have ascribed the exacerbation to his amantadine and “made him go cold turkey.” Amantadine was reinstated, states P12, only when she reminded staff that the abrupt withdrawal of amantadine could aggravate PD and its mental manifestations. Other participants, such as P11, spoke of medication “omissions for several days” in hospital, even though it is “imperative that none of the Parkinson's medications be halted.” No participants reported a decision to suddenly discontinue any PD medications themselves.

### 3.2. Instructions Wrong, Vague or Misread

P16 stated that for two years her community pharmacy, despite “a lot of the staff changing all the time,” had dispensed two PD medications (sinemet and entacapone) to her with the labelled instruction: “Take six tablets once daily as directed.” Recognising this instruction as a dangerous mistake, she reported instead taking one tablet of each medicine every three hours.

Other wrong instructions were said to have been given in non-neurological, hospital wards through the mischarting of dosing frequencies. According to P20, herself a practice nurse, this error led to her father with PD receiving doses at wrong times over two days. P12 also described how “the charting would change (for her husband with PD) … they would have 8.00, 8.30 … and I would say, “He is supposed to get his pergolide on a full stomach.” “Oh, no, no, it's charted for …”.

Othertimes, information was not wrong but misread: “She (the House Surgeon) went back and said, “The nurse has not read it (the chart). It is 1300 hours, not 3.00 at all”” (P12). P4 similarly indicated that hospital staff “just glanced down” at her partner's chart, getting “in the routine of giving him one without checking it thoroughly.” She reported that the neurologist had assured her that the chart was correct, and that “human error” accounted for her daily observation that the sinemet dosage was short and given “late, anything up to three-quarters of an hour.” 

In community settings, however, the problem was sometimes the vagueness of dispensing instructions. P19 reported how her family had misunderstood instructions to take a medication “four times a day.” They had thought this indicated a need “to time the (Pd) medicine to four tablets over 24 hours” even though this led to “big lows and big highs” and interrupted their sleep for several months. On the basis of the advice of the prescriber, the Parkinson's Society field officer explained to the family that “you need to give them during the daytime.”

### 3.3. Devaluation of the Lay Role

Several participants suggested that hospital staff wanted to take control of the PD medications and did not seek or respect the insights or perspective of the person with PD or their family. According to P20, timing errors could have been avoided “if they (hospital staff) had only asked me (the caregiver)—I had the latest script.” And when patients or caregivers offer information, “nobody listens, like you try and tell them something and they think they know better all the time.” This was despite people with PD having experience of what worked best for *them*: “… if you are taking them everyday, you know when you need to take them; your body tells you” (P5), so … “it is not really a sort of arranging it at the same time every day” (P6).

It was felt that staff commitments to change the timing could not be relied upon: “they would say, “oh yes, we will do that tomorrow” but it never happened” (P5). P8 concurred: “They are good at talking on, rather than listening.” P4 added, “they did not like being corrected, any of them … I felt that they would have been happier if I had not been there and chased them up on times.” Indeed, some staff were perceived to be patronising. P4 said that her partner with PD “was dismissed” by a nurse who “was very abrupt and ignored him and virtually walked off.” This was despite—and perhaps contributed to by—his PD making him “slightly slower to respond” and asking of “him quite a bit of courage to speak out.” Another caregiver, P12, reported that “they will talk to him and they will ignore me.”

### 3.4. Lack of Both Professional Knowledge and Caring Behaviour

Participants suggested that staff “do not always understand the way the (PD) medications work” (P6) and “were not aware, I think, of the need for Parkinson people to have their medication at a given time” (P9); they “regard the times as a suggestion … an indication of when you might get them” (P12). P6 acknowledged that she was like them until she developed PD: “I nursed a lot of them in the rest homes and hospitals that I have worked in and I was not aware really of the importance.” P9 added that staff ‘admitted afterwards that it was different for them, having a Parkinson patient … and a big learning curve”.

“Part of the problem,” suggested P8, “is they are short staffed … they are rushing round all over the place … and medication times fall by the way side.” As a consequence, added P12, “if they were busy, it (the charted times) did not matter… and there was no check whether he (her husband) took the pill”. P4, however, questioned the attribution to staff shortages: “there were numerous staff standing around … you could hear conversation and it was not medical conversation … it was more casual talk … (so) it seems that they could not have given a damn.”

### 3.5. Lay Forgetfulness

Timing errors were commonly also ascribed to lay error, through people with PD, and their caregivers: forgetting to administer the PD medication on time, for example because “I am busy doing something” (P8); forgetting to use what reminds them (e.g., the timer) when to take their medication; and forgetting “whether I have taken it or not” (P3). These memory errors were reported to take place only occasionally. Consequences included taking late or extra doses of sinemet to manage motor fluctuations (and then adjusting the timing of the remaining doses) but tending to miss the forgotten doses of other, less potent antiparkinson medications: “I often do forget the ropinirole and that is not such an issue … I just skip that dose” (P5) and “I would suddenly think, “Oh, I forgot the amantadine and the pergolide and it is now 3.30 p.m.; there is no point in having it”” (P12).

## 4. Discussion

This qualitative paper has reported perspectives held by people with PD, and their caregivers, around factors contributing to possible medication timing errors for PD. Across hospital and community settings, five sets of factors have been identified, which add to the small amount of published research on medication errors in PD. These results are likely to be transferable to health systems similar to New Zealand, since they confirm anecdotal experience internationally as well as policy and program responses that have been introduced in the absence of research evidence. 

PD medications were not given on time when abruptly withdrawn. The United Kingdom's National Institute for Health and Clinical Excellence (NICE) guideline for managing PD has highlighted the dangers of staff suddenly stopping antiparkinson medications [17]. Our paper has illustrated why a reduction in treatment with a medication such as amantadine must be gradual. Sudden withdrawal can exacerbate the PD, producing, or worsening, cognitive manifestations such as hallucinations. 

Participants in this study described how instructions for dispensing sometimes appeared wrong or vague. Their reports are consistent with research on medication labelling errors, such as wrong directions, by community pharmacies [18–20], and on instructions for medication-taking that can be “awkwardly phrased, vague, and unnecessarily difficult” [21]. For example, P19 described how her family had responded to a labelled instruction to administer PD medication “four times a day”. The family had interpreted this to mean a 24-hour period, which was not what the prescriber had intended. However, it was reasonable for the family to believe that timing adherence is not based only on waking hours because “smoother symptom control results from continuous, rather than pulsatile, drug delivery” [3]. 

The often high complexity of the medication regimen therefore increases the need for clear instructions on when to take the medication. Yet the regimen complexity and the low health literacy of many lay people increase the risk that vagueness will prevent them understanding the intended meaning of labelled instructions [10]. Misreading of instructions for the timing of PD medication was also reported in this study to characterise hospital and care home records and behaviour. This finding is consistent with the “Get it on time” campaign [8] and with calls for physician and pharmacist review of patient ward charts [22]. 

Some participants reported struggling to have their views heard and valued in hospitals. Staff were frequently said not to respect the ability of lay people to choose to manage their own medications for PD (even though the NICE guideline supports this possibility) [17]. Areas of disagreement over the lay role, and medication timing errors in PD by both professionals and lay people, create an opportunity in all health care settings for both parties to work together [23].

Many participants reported that people with PD, and their caregivers, occasionally forget to take their PD medication on time. The reasons they gave, such as busyness, are consistent with their capacity to err, through lapses in prospective memory [24]. Episodic information on the timing of doses is, itself, subject to age-related memory loss [25]. However, acting against forgetfulness are factors such as wearing off and “on-off” phenomena, and the perceived importance by lay people of administering PD medication on time to relieve motor symptoms.

## 5. Strengths and Limitations

This study permitted people with PD, and their caregivers, to represent their experience of issues around PD medication mistiming and advocate for change. Lay people expect, and are highly motivated to avoid, unsafe care delivery [23] and are in the best position to assess and comment on their own contributions to error. Their perspectives on medication timing errors can supplement those of professionals; elucidate lay attitudes towards, and behaviour in, the health system; and contribute to system-wide approaches to medication error management.

Semistructured interviews by telephone were able to elicit these perspectives in a manner that balanced the need for a flexible yet systematic approach to data collection. Interviewing by telephone also collected data cost-effectively and quickly from a geographically dispersed, national sample. Telephone interviews are feasible with most people with PD [26, 27] and the opportunity to involve proxies minimised the loss of people with PD who find telephone use challenging. 

However, this study has limitations. The sample is small, but it provides in-depth information. Also, it “is biased; it must be biased” [28] since purposive sampling was used to select the most information-rich, lay participants. These are the participants who can best represent the experience of perceived medication errors by health professionals. Unlike probability sampling, purposive sampling cannot therefore represent the background population from which participants were selected. We have, however, attempted to provide sufficient information for readers to decide whether our study findings are transferrable to their own settings.

 In addition, when errors in medication timing in PD become clinically important is not always clear [3]. Participants' reports in this study were subject to recall bias and their nonclinical knowledge might have restricted their ability to identify or attribute medication timing errors. No attempt was made to verify the reports or assess cognitive function; however, ambulatory [29] and hospitalised patients have the capacity to recognise medication errors [23]. Their perspectives also define what is real to them. Another limitation of our study is that the proxy reports may differ from the self-reports of the people with the medical condition [30]. Errors in treatment timing initiation for PD were outside the scope of this study.

## 6. Conclusions

The lay perspectives reported in this paper highlight opportunities from lived experience to reduce possible, avoidable errors in the timing of medication ingestion for PD, and attrition of health gain and quality of life. Further research is needed to validate lay reports against external evidence including the observations of professionals, and medical records. However, the narratives reported in this study indicate changes that may improve the timing of PD medication. Such changes include effective communication and shared respect for the capacity of professionals and lay people to co-manage PD medication delivery in hospital and community settings. People with PD, and their caregivers, can contribute to this partnership because they have experience of meeting their own daily needs to manage PD, which professionals do not have. Their lay knowledge can and should be enhanced through a therapeutic alliance with health professionals, which can reduce the risk that each party will err alone. This shared care approach resonates with the NICE guideline [17] and other United Kingdom initiatives, such as the expert patient programme [31]. Change, however, requires a culture shift. Progress will be halting until professionals recognise the capacity of lay people to be involved in, and share responsibility for, their PD care in diverse settings. 

## 7. Competing Interests

Deirdre O'Sullivan is the National Director of Parkinson's New Zealand, which is the funder of this research and the organisation through which participants were recruited.

## Figures and Tables

**Table 1:** Participant attributes.

Participant	Proxy	Relationship to person with PD*		Person with PD		
			Age group	Sex	Self-rated PD severity	Years with PD diagnosis
1	No		<65	Male	Mild	5
2	No		<65	Male	Severe	12
3	No		<65	Male	Mild	9
4	Yes	Partner	<65	Male	Moderate	10
5	No		<65	Female	Mild	4
6	No		<65	Female	Mild	13
7	No		65–74	Male	Moderate	18
8	No		65–74	Male	Moderate	14
9	No		65–74	Male	Moderate	10
10	Yes	Wife	65–74	Male	Moderate	3
11	No		65–74	Male	Moderate	12
12	Yes	Wife	65–74	Male	Moderate	10
13	No		65–74	Male	Moderate	14
14	No		65–74	Female	Moderate	11
15	Yes	Husband	65–74	Female	Moderate	8
16	No		65–74	Female	Moderate	8
17	Yes	Partner	75+	Male	Moderate	12
18	No		75+	Male	Moderate	29
19	Yes	Daughter	75+	Female	Severe	7
20	Yes	Daughter	75+	Male	Moderate-severe	11

*PD = Parkinson's disease.
